# Morphological and vascular characteristics of the optic nerve head of normal guinea pigs

**DOI:** 10.1038/s41598-022-04911-x

**Published:** 2022-01-18

**Authors:** Lei Guo, Jun Tao, Yang Tong, Shichao Chen, Xin Zhao, Rui Hua

**Affiliations:** 1 Ophthalmology and Optometry Centre, The First Hospital of China Medical University, Shenyang, China; 2 Department of Ophthalmology, Shenyang Xingqi Eye Hospital, Shenyang, China; 3 Department of Ophthalmology, The 4th People’s Hospital of Shenyang, Shenyang, China; 4 Department of Ophthalmology, The First Hospital of China Medical University, No. 155, Nanjingbei Street, Heping District, Shenyang, 110001 Liaoning Province China; 5 Macular Disease Research Laboratory, Department of Ophthalmology, West China Hospital, Sichuan University, Chengdu, China

**Keywords:** Eye manifestations, Optic nerve diseases

## Abstract

The morphological and vascular characteristics of the optic nerve head (ONH) of normal guinea pigs have not been fully recognized. Therefore, we aimed to investigate them using optical coherence tomography (OCT) and optical coherence tomography angiography (OCTA). We measured the refractive error, axial length, and intraocular pressure (IOP) and performed OCT and OCTA of the ONH of 3- and 4-week-old tricolour guinea pigs. A total of 208 right eyes from 208 normal guinea pigs were examined. The refractive error (both myopic and hyperopic) of the 3-week group was significantly higher than that of the 4-week group (p < 0.001), the IOP of the 3-week group was significantly lower than that of the 4-week group (p = 0.014), and the circumpapillary retinal nerve fibre layer (cpRNFL) of the 3-week group was significantly thicker than that of the 4-week group (p = 0.048). There were no significant differences in the average vessel area, vascular density, total number of junctions, total vessel length, total number of endpoints, and vascular diameter between the two groups. However, an age-adjusted linear regression analysis revealed that the total vessel length was positively associated with the cpRNFL thickness (p = 0.024) and negatively associated with IOP (p = 0.016). This is the first report on morphological and vascular characteristics of the ONH in normal guinea pigs based on in vivo OCT and OCTA imaging and quantification of ONH parameters. These results may contribute to further research on myopia using guinea pig models.

## Introduction

The optic nerve head (ONH) is an important structure involved in the pathogenesis of myopia^1^. Guinea pigs have been increasingly used as experimental models of myopia^2^. In an in vitro study, Ostrin et al. reported the structural anatomy of the retina and ONH in guinea pigs^3^. Haematoxylin and eosin-stained sections showed retinal ganglion cell axons organised into fascicles in the prelaminar and laminar regions of the ONH, and scanning electron microscopy revealed a well-defined lamina cribrosa with radially oriented collagen beams^3^.

Optical coherence tomography (OCT) and OCT angiography (OCTA) are recent technological advancements for the diagnosis, treatment, and follow-up of several retinal diseases due to their ability to capture high-resolution characteristics of the retina within seconds^4^. In our previous study, we found that a U-shaped neural canal and a lower ratio of Bruch membrane opening to minimum rim width were early indicators of retinal nerve fibre layer (RNFL) defects in pre-perimetric glaucomatous eyes of glaucoma patients with normal circumpapillary RNFL (cpRNFL) thickness in OCT images^5^. Jnawali et al. observed that histological sections of all the retinal layers in guinea pigs were consistent with their OCT images, and hyper-reflective regions of the OCT images corresponded to the retinal pigment epithelium, inner/outer segment junction, outer plexiform layer, inner plexiform layer, and RNFL^2^. However, the anterior lamina cribrosa surface cannot be visualised in OCT images because the thick RNFL and vascular sheath obscure the ONH, which decreases the penetrance of the scanning laser. The reliability of OCTA for measuring choroidal thickness and choroidal blood perfusion has been verified in guinea pigs^6^. In addition, OCTA-related parameters—including vascular density, number of vessel junctions, total vessel length, and number of vessel endpoints—have been introduced to assess choroidal neovascularisation in rat models; these parameters showed significantly higher values for the intermediate and deep vascular plexi of choroidal neovascularisation when obtained by OCTA than when obtained by fluorescein angiography^7^. Similar to the ONH in humans, the ONH of guinea pigs also contains vascular tissue. Protruding vascular tufts in the ONH have been reported in guinea pigs^2^; however, little is known about the normal imaging characteristics of their blood perfusion and OCTA parameters. Quantification of such parameters in normal guinea pig models is critical for characterising vascular structural changes expected to occur in conditions like myopia.

In the present study, we aimed to investigate and set the normal ranges for both morphological and blood perfusion characteristics of the ONH in normal guinea pigs using OCT and OCTA and investigate the factors influencing blood perfusion in the ONH. We conducted this study to provide normative data for guinea pigs considered potentially valuable models for myopia.

## Methods

### Animals

Our study was conducted on 3- and 4-week-old tricolour guinea pigs stratified by their age. Three-four week guinea pigs are successful models for myopia^6^. Because there are no statistically significant differences between the refractive and biometric measurements (intraocular pressure [IOP] and axial length [AL]) in the right and left eyes^2,8^, only the right eye was selected for the study in all groups. Guinea pigs with congenital cataracts, corneal abnormalities, or other ocular diseases that may affect vision were excluded at the baseline. All animal care and examinations in this study conformed to the ARVO Statement for the Use of Animals in Ophthalmic and Vision Research, and all experimental protocols were approved by the Animal Care and Use Committee of China Medical University. The study is reported according to the ARRIVE guidelines (https://arriveguidelines.org).

### Parameter measurements

Initially, IOP was measured in all eyes using a tonometer (Icare Finland Oy, Vantaa, Finland). The IOPs of the guinea pigs have been demonstrated to undergo diurnal variations, peaking in the early morning and decreasing across the day^3^; therefore, measurements were always taken at the same time of day (afternoon) to prevent confounding effects of diurnal rhythms. Three sets of six measurements were recorded, and the average IOPs were calculated. Guinea pigs do not require anaesthesia for IOP measurements; therefore, anaesthesia-induced alterations in IOP can be prevented^2^.

And then, topical tropicamide eye drops were administered three times at an interval of 5 min for cycloplegia, and assessments were performed 30 min later. Cycloplegic refractive error was measured using a streak retinoscope (YZ-24, Suzhou Liuliu, China).

The ONH was assessed using spectral-domain OCT (Cirrus HD-OCT, Zeiss, Germany) in the ONH mode. We designed the examining pallet for animal studies, and the focus plane of OCT was adjusted for imaging of the ONH without an animal customized cornea lens for OCT examination. The positions of the eyes of the guinea pigs were controlled and fixed by regulating the stem fixed in the mouth. All the OCT parameters originated from the instrument’s software except for calculating the parameters of OCTA, which were achieved by AngioTool software11 (a custom program outside of the Cirrus). The analysed parameters were average cup-to-disc ratio, cpRNFL thickness (μm, the RNFL of the circle with a diameter of 3.4 mm centred at the disc), and cup volume (mm^3^). Blood flow signals of the ONH were also recorded on OCTA (Cirrus HD-OCT, Zeiss, Germany) within the 6 × 6-mm range. SD-OCT and OCTA measurements were performed under general anaesthesia (intraperitoneal anaesthesia) with 1% pentobarbital sodium (0.33 ml/100 g).

Finally, when the pupil returned to normal, AL was measured using an A-scan ultrasound (Aviso, Quantel Medical Inc., Cournon-d’Auvergne, France; probe diameter, 5 mm; ultrasonic resolution, 0.01 mm; ultrasonic transmission frequency, 11 Hz) under topical anaesthesia.

### Analysis of OCTA images

**Figure 1:**
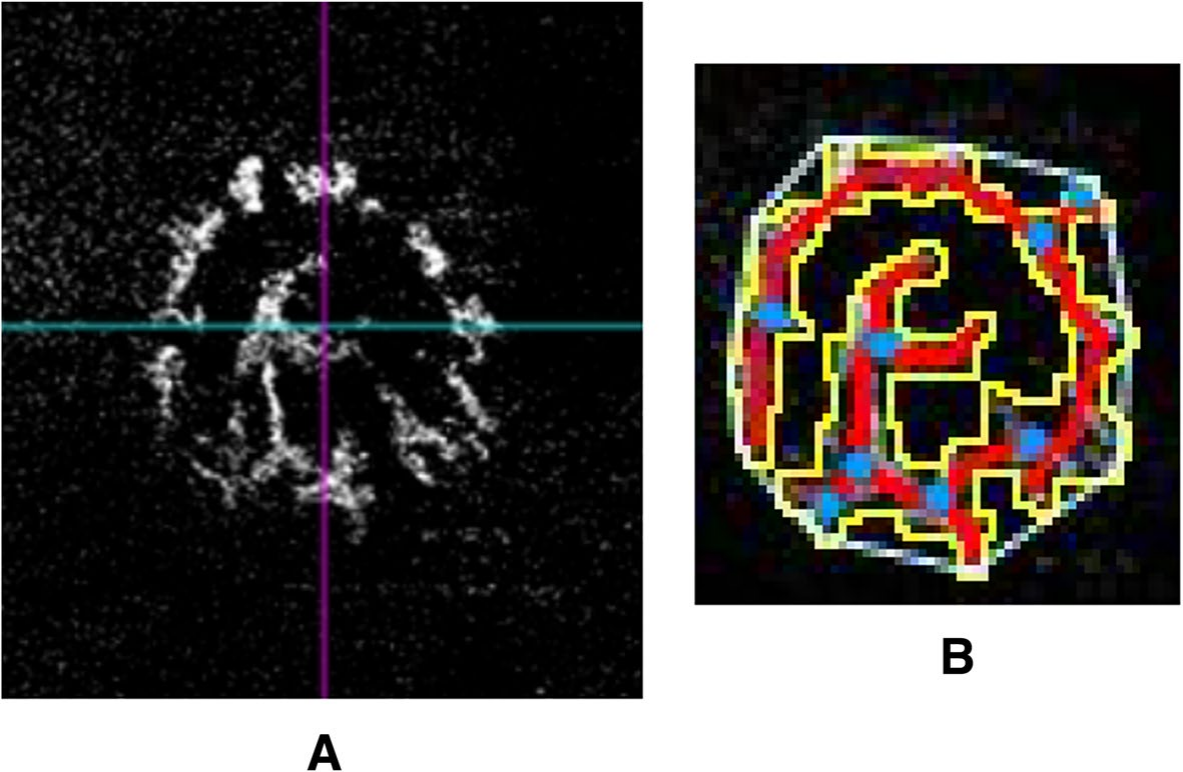
Analysis of OCTA parameters of the ONH at the age of 4 weeks using AngioTool software. (**A**) Retinal avascular layers segmentation mode was applied to the ONH and selected by the inbuilt software of OCTA and were subsequently imported to the AngioTool software. (**B**) The AngioTool software automatically measured the explant area (area within blue circles), vessel area (area within yellow circles), vascular density, total number of junctions (blue dots), junction density, total vessel length (red lines), average vessel length, and the total number of endpoints of vessels. *OCTA* optical coherence tomography angiography, *ONH* optic nerve head.

Guinea pig eyes do not have any retinal vessels outside the ONH region, which is proven by fundus angiography Phoenix-OCT fluorescence angiography in our previous unpublished study (Glaucomatous abnormalities following scleral cross-linking in form-deprivation myopic guinea pig eyes [under consideration]). In this study, the blood flow signal maps of ONH were obtained using the OCTA device, and the retinal avascular layer mode was selected. The retinal avascular layer mode of the Cirrus OCT is used for automatic segmentation of the retinal vasculature. However, no automatic segmentation mode for the ONH is available for OCTA, and we used this retinal avascular layer mode to analyse the vasculature of the ONH based on our observation. Subsequently, these target images were imported to the AngioTool software^9^, which automatically measured the vessel area, vascular density, total number of junctions, total vessel length, and total number of endpoints of vessels of the ONH (Fig. 1)^10^. The vascular diameter was defined as the vessel area divided by the total vessel length. We consider that the nutrient vessels within the ONH, which was showed by OCTA, may be central retinal vessels and their capillary system. However, no related histopathological studies have been reported until now. Nevertheless, we believe that this vascular system was responsible for the nutrition of the optic nerve, which may play an important role in the progression of myopia.

### Statistical analyses

All data were analysed using SPSS 24.0 statistical software (IBM, SPSS Statistics, Version 24, SPSS Inc., Chicago, USA). The data are expressed as mean ± standard deviation. Independent samples t-test was used for pair-wise comparisons of AL, IOP, refraction, and OCT- and OCTA-related parameters for the 3- and 4-week old groups. Pearson’s test was used to determine the correlations between vascular density, total vessel length in the ONH, and all other parameters. The factors influencing vascular density and total vessel length of the ONH were determined using linear regression analysis. A p-value of < 0.05 was considered statistically significant.

## Results

### General information

A total of 208 right eyes from 208 normal guinea pigs were included, which comprised 83 3-week-old (3-week group) and 125 4-week-old (4-week group) guinea pig eyes. The average AL, refractive error, and IOP were 7.68 ± 0.21 mm, + 2.38 ± 2.10 D, and 17.4 ± 3.04 mmHg, respectively. No significant difference in AL was found between the 3-week and 4-week groups (7.67 ± 0.19 mm vs. 7.69 ± 0.22 mm, t = 0.432, p = 0.666). The refractive error in the 3-week group was significantly larger than that in the 4-week group (+ 3.02 ± 1.27 D vs. + 1.96 ± 2.37 D, t = 3.710, p < 0.001). The IOP in the 3-week group was significantly lower than that in the 4-week group (16.7 ± 3.19 mmHg vs. 17.8 ± 2.88 mmHg, t = 2.483, p = 0.014).

### Morphological parameters of the optic disc based on OCT

**Figure 2:**
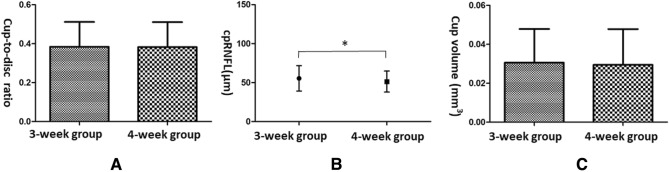
Morphological parameters of the optic nerve head in normal guinea pigs. (**A**) Cup-to-disc ratio: 0.38 ± 0.128 (3-week group) and 0.38 ± 0.128 (4-week group). (**B**) Thickness of circumpapillary retinal nerve fibre layer: 55.4 ± 16.27 μm (3-week group) and 51.3 ± 13.45 μm (4-week group). (**C**) Cup volume: 0.031 ± 0.0173 mm^3^ (3-week group) and 0.029 ± 0.0184 mm^3^ (4-week group). *p < 0.05.

The cup-to-disc ratio, cpRNFL thickness, and cup volume of the 208 eyes were 0.38 ± 0.13, 53.0 ± 14.74 μm, and 0.030 ± 0.018 mm^3^, respectively. The cpRNFL thickness in the 3-week group was significantly larger than that in the 4-week group (55.4 ± 16.27 μm vs. 51.3 ± 13.45 μm, t = 1.993, p = 0.048). However, no significant differences were found in the cup-to-disc ratio and cup volume between the 3-week and 4-week groups (Fig. 2).

### Blood perfusion in the ONH based on OCTA

The vessel area, vascular density, total number of junctions, total vessel length, total number of endpoints, and vascular diameter were 924.64 ± 226.33 pixels, 48.86 ± 7.12%, 6.84 ± 3.54, 180.70 ± 49.44 pixels, 7.93 ± 3.20, and 5.25 ± 1.03 pixels, respectively. There were no significant differences in any of these parameters between the 3-week and 4-week groups (Table 1).

**Table 1:** Blood flow parameters of the optic disc head based on OCTA.

	N	Vessel area (pixels)	Vascular density (%)	Total number of junctions (N)	Total vessel length (pixels)	Total number of endpoints (N)	Vascular diameter (pixels)
3-week group	83 eyes	920.63 ± 227.994	48.44 ± 7.493	6.6 ± 3.39	180.81 ± 49.427	7.6 ± 2.88	5.24 ± 1.167
4-week group	125 eyes	927.31 ± 226.097	49.15 ± 6.871	7.0 ± 3.64	180.63 ± 49.640	8.2 ± 3.38	5.26 ± 0.927
		t = 0.208, p = 0.835	t = 0.700, p = 0.485	t = 0.897, p = 0.371	t = 0.026, p = 0.979	t = 1.394, p = 0.165	t = 0.118, p = 0.906

### Factors influencing the vascular density and total vessel length of the ONH

The vascular density of ONH was positively correlated with vessel area (r = 0.508, p < 0.001), total number of junctions (r = 0.348, p < 0.001), total vessel length (r = 0.265, p < 0.001), and vascular diameter (r = 0.241, p < 0.001) and negatively correlated with the total number of endpoints (r =  − 0.460, p < 0.001). However, an age-adjusted linear regression analysis revealed that vascular density was positively associated with vessel area (B = 0.036; 95% confidence interval [CI] 0.029 to 0.043; p < 0.001) and the total number of junctions (B = 1.363; 95% CI 1.096 to 1.629; p < 0.001) and negatively associated with the total vessel length (B =  − 0.172; 95% CI − 0.211 to − 0.132; p < 0.001), total number of endpoints (B =  − 1.418; 95% CI − 1.576 to − 1.260; p < 0.001), and vascular diameter (B =  − 0.647; 95% CI − 1.490 to 0.197; p < 0.001).

The total vessel length of ONH was positively correlated with vessel area (r = 0.870, p < 0.001), vascular density (0.265, p < 0.001), total number of junctions (r = 0.853, p < 0.001), and total number of endpoints (r = 0.257, p < 0.001). However, it was negatively associated with the vascular diameter of ONH (r =  − 0.469, p < 0.001). cpRNFL thickness (r = 0.110, p = 0.113) and IOP (r = 0.076, p = 0.273) did not show any relationships with total vessel length. However, an age-adjusted linear regression analysis revealed that the total vessel length was positively associated with vessel area (B = 0.166; 95% CI 0.155 to 0.177; p < 0.001), total number of junctions (B = 4.413; 95% CI 3.632 to 5.194; p < 0.001), and cpRNFL thickness (B = 0.110; 95% CI 0.015 to 0.205; p = 0.024) and negatively associated with vascular density (B =  − 1.622; 95% CI − 1.973 to − 1.271; p < 0.001), total number of endpoints (B =  − 2.319; 95% CI − 3.002 to − 1.636; p < 0.001), vascular diameter (B =  − 13.209; 95% CI − 14.920 to − 11.497; p < 0.001), and IOP (B =  − 0.611; 95% CI − 1.108 to − 0.114; p = 0.016).

## Discussion

Guinea pigs have become increasingly popular models for studying human myopia^3^. Therefore, it is important to have data on the normal development of the eyes of guinea pigs. The main goals of this study were to set the normal ranges for both morphological and vascular parameters of the ONH in normal guinea pigs and analyse the factors influencing blood perfusion in the ONH.

Our results showed that no significant differences were found in the cup-to-disc ratio, and cup volume between the 3-week and 4-week groups. However, as no related analyses have been reported, we were unable to compare them to any corresponding reference values. RNFL thickness is an important clinical diagnostic measure for glaucoma^11^. In our study, the cpRNFL thickness of the 3-week group (55.4 ± 16.27 μm) was significantly larger than that of the 4-week group (51.3 ± 13.45 μm). Jnawali et al. reported that the RNFL thickness of the 2.5-year-old pigmented guinea pigs was 59.2 ± 4.5 μm^2^, which was greater than our result.

OCTA and AngioTool software have been successfully used in clinicopathological studies with high accuracy and reproducibility. For example, Zimmermann et al. used AngioTool software to assess the performance of OCTA for imaging of the vascular network in the SVP and DVP and concluded that OCTA is a helpful imaging tool for non-invasive and in vivo imaging of the vascular plexus in mice. It may offer advantages over fluorescein angiography and confocal microscopy especially for imaging the deep vascular plexus^10^. Similarly, Liu et al. successfully extracted the retinal vasculature and quantified the retinal vessel branch points, vascular area, and vessel lengths using the AngioTool and concluded that simultaneous non-invasive analysis of the retinal vessels and neurons by OCTA and OCT may provide a novel approach for characterising retinal ischemia accompanied by neurovascular coupling^12^. Guinea pigs were used in the present study, and the structure and vasculature of ONH, instead of the retina, were analysed. No previous study has reported on this, and our study focused on the vasculature of the ONH. The vessel area, vascular density, total number of junctions, total vessel length, total number of endpoints, and vascular diameter were 924.64 ± 226.33 pixels, 48.86 ± 7.12%, 6.84 ± 3.54, 180.70 ± 49.44 pixels, 7.93 ± 3.20, and 5.25 ± 1.027 pixels, respectively. It has been reported that the vascular density within the Bruch membrane opening of rhesus monkeys was 42.7% (10 mmHg), 43.5% (20 mmHg), and 43.3% (30 mmHg)^13^.

Age-adjusted linear regression analysis revealed that the cpRNFL thickness is positively associated with total vessel length, and IOP is negatively associated with total vessel length. Manalastas et al. reported that the thicknesses of cpRNFL and the macular ganglion cell complex are more strongly associated with the vascular density of the ONH of glaucoma patients, glaucoma suspects, and healthy individuals^14^. Moreover, age-adjusted linear regression analysis revealed that vascular density is positively associated with vessel area and the total number of junctions and negatively associated with total vessel length, total number of endpoints, and vascular diameter.

Our results revealed that the AL, refractive error, and IOP were 7.68 ± 0.21 mm, + 2.38 ± 2.10 D, and 17.4 ± 3.04 mmHg, respectively. Jnawali et al. reported that the mean AL was 10.00 ± 0.12 mm for guinea pigs aged 2.5 years^2^. In addition, two previous studies on normal guinea pigs (age unknown) reported a mean IOP of 16.5 ± 3.2 mmHg for 100 normal guinea pigs^15^ and 18.27 ± 4.55 mmHg for 31 normal guinea pigs^16^. In our study, the IOP of the 3-week group was significantly lower than that of the 4-week group (16.7 ± 3.19 mmHg vs. 17.8 ± 2.88 mmHg). Similarly, Jnawali et al. reported that IOPs showed an early minimal increase that appeared to stabilise subsequently with age^2^.

Our study showed that the refractive error of the 3-week group was significantly greater than that of the 4-week group (+ 3.02 ± 1.27 D vs. + 1.96 ± 2.37 D). Jnawali et al. also reported that the young guinea pigs were hyperopic, with a mean spherical equivalent refraction of + 3.50 ± 0.77 D^2^.

No difference was found in AL between the 3-week and 4-week groups (7.67 ± 0.19 mm vs. 7.69 ± 0.22 mm). Similarly, Zhou et al. reported that the increase in AL was relatively rapid during the first week, followed by minimal changes during the next 3 weeks and rapid increments after 5 weeks^8^. The emmetropisation process in guinea pigs is mainly related to the increase in the vitreous chamber length, and the axial development of the vitreous chamber in guinea pigs appears to be associated with posterior scleral tissue growth^8^.

This study has limitations. First, we used the Cirrus OCT, which is designed for much larger human eyes, to assess the ONH of the eyes of the guinea pig. Second, we did not measure the refractive powers of the guinea pigs at birth. These limitations should be addressed in further research.

Nevertheless, this is the first report on the morphological and vascular characteristics of the ONH in normal guinea pigs based on in vivo OCT/OCTA imaging and quantification of ONH parameters. Hence, our results also corroborate the report that the maturation of guinea pig eyes continues in the early stages of life, and they may provide excellent models for myopic investigation.

## Data Availability

All data are included in the manuscript.
